# The association between body mass index and the risk of different gastrointestinal cancers: A protocol for an overview of systematic reviews

**DOI:** 10.1097/MD.0000000000013181

**Published:** 2018-11-09

**Authors:** Xueni Ma, Ya Gao, Jipin Li, Dekui Zhang, Jinhui Tian

**Affiliations:** a The Second Clinical Medical College of Lanzhou University; b Evidence-based Medicine Center, School of Basic Medical Sciences, Lanzhou University; c Department of Gastroenterology, Lanzhou University Second Hospital, Lanzhou, China.

**Keywords:** body mass index, gastrointestinal cancers, incidence, mortality, obesity, overweight

## Abstract

**Background:**

Changes in our lifestyle over the past few decades have led to a significant increase in the worldwide prevalence of both overweight (defined as a body mass index [BMI]≥25 kg/m^2^) and obesity (BMI≥30 kg/m^2^), thus leading to numerous harmful consequences for an individual's health. Several meta-analyses support the link between obesity and different gastrointestinal cancers, but substantial heterogeneity exists between studies. We will synthesize published systematic reviews to evaluate the association between body mass index (BMI) and the incidence and mortality of different gastrointestinal cancers.

**Methods:**

PubMed, EMBASE, and the Cochrane Library will be systematically searched for systematic reviews, meta-analyses, and pooled analyses in July, 2018. Two review authors will independently screen titles and abstracts for relevance, assess full texts for inclusion, carry out data extraction, and appraise methodological quality using AMSTAR checklist and reporting quality using PRISMA statement. The association between BMI and different gastrointestinal cancers will be estimated by computing the pooled relative risk (RR) and its 95% confidence interval (CI), which will be calculated from the adjusted RR, odds ratio, or hazard ratio, and 95% CI offered in the studies. Heterogeneity between studies will be assessed with the *I*^*2*^ statistic as a measure of the proportion of total variation in estimates that is due to heterogeneity, where *I*^*2*^ values of 25%, 50%, and 75% correspond to cut-off points for low, moderate, and high degrees of heterogeneity. The random effects model will be used as the pooling method when significant heterogeneity existed and the fixed effect model will be used when no heterogeneity was observed. Possible publication bias will be tested by Begg and Egger test.

**Discussion:**

This overview of systematic reviews will provide an accessible, comprehensive synthesis with which to inform clinicians and the development of guidelines for the management of the individuals with high BMI.

**Ethics and dissemination:**

Only published secondary data will be used in this study, and therefore ethics approval is not required.

**PROSPERO registration number:** CRD42018107334.

## Abstract 2

Supplemental Digital Content is available in the text

## 1 Introduction

Cancer has been the major cause of death in most regions of the world.^[1]^ Gastrointestinal cancers (GICs) have been common malignant tumors in humans, which have become a major health problem. Not to mention, the rates of incidence of the esophagus, stomach, and colorectal cancers are 3, 12, and 1.5 cases per 100,000 population.^[2]^ Gastric cancer (GC) has been the second highest cause of cancer-related mortality, and colorectal cancer (CRC) has been the third most fatal malignancy worldwide.^[3,4]^ Thus, GICs lead to huge economic burden worldwide, especially in Asian countries.^[5]^ On the other hand, a WHO report ^[6]^ indicated that overweight (body mass index [BMI]: 25.0–29.9 kg/m^2^) and obesity (BMI ≥30.0 kg/m^2^), which are defined as abnormal or excessive fat accumulation has more than doubled in the past thirty years. Approximately 2.3 billion adults are overweight and more than 700 million are obese globally.^[7]^ And the number of overweight and obese people has risen from approximately 857 million in 1980 to 2.1 billion in 2013.^[8]^

The association between body mass index and cancer has been extensively studied during the past several decades, but substantial heterogeneity exists between studies.^[9]^ The first meta-analysis was conducted back in 2008 by Renehan et al^[10]^ in which the authors reported associations between BMI and 20 cancer types. Specifically, they reported that higher BMI was associated with an increased risk of the following 7 types of cancers in both males and females: thyroid, kidney, colorectal cancers, esophageal adenocarcinoma, multiple myeloma, leukemia, and non-Hodgkin lymphoma. Since 2008, a large number of high-quality meta-analysis have been published to investigate the relationship between BMI and the risk of several cancers further.^[7,11,12]^ Among these cancers, gastrointestinal cancers, including liver cancer, gallbladder cancer, colorectal cancer, and pancreatic cancer were largely reported. In addition, Kyrgiou M et al^[13]^ conducted an umbrella review of the literature, in which the authors found strong evidence to support the positive association between obesity and 11 of the 36 cancer sites and subtypes that they examined, predominantly comprising cancers of the digestive organs and hormone-related malignancies in women. However, the reported associations may be causal, but they may also be flawed, as inherent study biases such as residual confounding and selective reporting of positive results may exaggerate the effect of obesity on cancer.^[14–17]^

Furthermore, according to our knowledge, there was no study that specifically evaluates the association between BMI and the risk of different gastrointestinal cancers. The purpose of this overview of systematic reviews is to synthesize the evidence evaluating the association between BMI and the risk of different gastrointestinal cancers. Specifically, the incidence of the cancer is the focus of this review. In addition, we aimed to quantify the risk of different cancers associated with an incremental increase in BMI.

## 2 Methods/Design

### 2.1 Protocol and registration

We will adhere to standards of the Preferred Reporting Items for Systematic Reviews and Meta-analyses (PRISMA) in reporting the findings of this review.^[18]^ The content of this protocol follows the PRISMA Protocols (PRISMA-P) recommendations.^[19]^ This review is registered with the International Prospective Register of Systematic Reviews (PROSPERO).^[20]^ The registration number is CRD42018107334.

### 2.2 Search for and identification of studies

Two authors will independently search the PubMed, Embase, and Cochrane Library from inception to July 21, 2018 for systematic reviews, meta-analyses or pooled analyses to identify relevant studies. Our search will be restricted to studies conducted in humans, and no restrictions will be imposed regarding the language in which the studies were published. The databases will be searched using search terms related to BMI, obesity, overweight, cancer, and meta-analysis. The literature search strategies will be reported in detail in Supplementary 1. In addition, reference lists of included studies will be manually searched for additional references.

### 2.3 Eligibility criteria

Studies that met the following criteria will be included in our overview of systematic reviews: systematic reviews, meta-analyses, or pooled analyses; the exposure of interest was BMI, overweight, or obesity defined by BMI; relative risk (RR), hazard ratio (HR), or odds ratio (OR) with its corresponding 95% confidence interval (95% CI) for BMI and the incidence or mortality of gastrointestinal cancers were included; the authors reported risk estimates (with 95% CIs) for at least 3 quantitative categories of BMI or sufficient information to calculate them; and reports of studies in which the association between BMI, obesity, or overweight and the incidence or mortality of cancers that contained any gastrointestinal cancers will be also included in the review. Studies were excluded if: publications were not full reports, such as conference abstracts and letters to editors; the estimates were presented without risk estimates and its corresponding 95% CI or other information that allowed calculation of data; protocols of reviews or methodological articles; or systematic reviews without meta-analysis.

### 2.4 Study selection and data abstraction

Literature search records will be imported into EndNote X7 (Thomson Corporation, CT) literature management software. Two review authors will independently screen the titles and abstracts of retrieved publications to identify potentially eligible studies for inclusion. The same 2 reviewers will retrieve full-text reports of potentially eligible studies and independently determine study inclusion or exclusion. Any disagreement will be resolved by a third reviewer.

Data will be extracted using a standardized data collection form. Two investigators will independently extract detailed information from each included article. Discrepancies will be resolved through group discussion with a third investigator. The following information will be extracted from each study: the first author of the publication, year of publication, features of the cancers, and the corresponding risk estimates (with 95% CIs). We will extract the risk estimates with the most adjustments and for the studies that reported the risk estimates of adjusted or not adjusted for publication bias; we extracted the former rather than the latter. Furthermore, we will extract the risk estimates calculated by a random-effects model if the outcome analyzed by the random-effects and fixed-effects model in the same study.

### 2.5 Quality assessment

We will assess the methodological quality of included studies using the Assessment of Multiple Systematic Reviews (AMSTAR) checklist. This checklist includes 11 items, with possible responses of “Yes” (item/question fully addressed), “No” (item/question not addressed), “Cannot answer” (not enough information to answer the question), and “Not applicable”. A score of 1 was attributed for each “yes” and “0” point for any other responses (“no,” “Cannot answer,” and “Not applicable”).^[21]^ Based on previous overviews, we will consider studies with a score between 0 and 4 to be of low quality, 5 and 8 to be of moderate quality, and 9 and 11 to be of high quality.^[22–23]^

The reporting quality of included systematic reviews will be assessed according to the PRISMA statement, which is a checklist of 27 items. To indicate the degree of compliance, each checklist item will be assigned one of the following 4 responses: “yes” for total compliance; “partial” for partial compliance; “no” for noncompliance; and “cannot answer” for limited information. The total score of reporting quality was obtained by summing “1” point for each “yes,” “0.5” for each “partial,” and “0” point for any other responses (“no,” and “cannot answer”).^[21]^ The quality assessment of the included studies will be performed independently by the 2 authors and the disagreements will be resolved through discussion to reach a consensus.

### 2.6 Statistical analysis

The association between BMI and gastrointestinal cancers will be estimated by computing the pooled RR and its 95% CI, which was calculated from the adjusted RR, OR, or HR and 95% CI offered in the studies. In this meta-analysis, HRs and ORs were deemed equivalent to RRs.^[24]^ We will define body mass categories using the following BMI categories: normal (BMI = 18.5–24.9 kg/m^2^), overweight (BMI = 25–29.9 kg/m^2^), and obesity (BMI≥30 kg/m^2^). We will also estimate the RR per 5 kg/m^2^ increase in BMI by regressing the natural logarithm of the RRs to the corresponding median values of BMI across exposure categories in each study, using the variance-weighted least-squares regression.^[25,26]^ This requires that the number of cases and person-time or non-cases for at least 3 quantitative exposure categories is known. We will assess heterogeneity between studies with the *I*^*2*^ statistic^[27]^ as a measure of the proportion of total variation in estimates that is due to heterogeneity, where *I*^*2*^ values of 25%, 50%, and 75% correspond to cut-off points for low, moderate, and high degrees of heterogeneity. The D-L random effects model^[26]^ will be used as the pooling method when significant heterogeneity existed and the M–H fixed effect model^[28]^ will be used when no heterogeneity was observed. Possible publication bias will be tested by Begg and Egger test^[29,30]^. All statistical analyses will be conducted using STATA, version 12.0 (College Station, TX). A 2-tailed *P* < .05 will be considered statistically significant.

## 3 Discussion

The effect of obesity on the incidence and mortality of cancer is well recognized.^[9,10,31]^ However, this will be the first overview of systematic reviews for the association between BMI and different gastrointestinal cancers. In addition, we aimed to quantify the risk of different cancers associated with an incremental increase in BMI. Furthermore, we hope to use the robust methodology to rigorously appraise and comprehensively synthesize published systematic review evidence. Overviews of systematic reviews generally provide the highest level of evidence for harms associated with treatment.^[32]^ However, Overviews of systematic reviews present several methodological challenges that should be considered.^[33–35]^ The biggest challenge may be using data more than once from individual primary studies without accounting for overlap may result in some primary studies being over-represented. As recommended, we will apply a priori criteria to select systematic reviews when there are multiple potential candidates.^[36]^

Our evidence synthesis will provide a new commentary on the current systematic review evidence for the association between BMI and the risk of different gastrointestinal cancers. It will provide an accessible, comprehensive synthesis with which to inform clinicians and the development of guidelines for the management of the individuals with high BMI. Furthermore, it is expected that this overview will encourage further research for those relationships for which systematic review evidence of high quality is currently insufficient or lacking.

## Acknowledgments

We express our thanks to the Evidence-based Medicine Center of Lanzhou University for the assistance with the design of the literature searches.

## Author contributions

XM, DZ, and JT conceived the idea for this study; DZ and JT designed the meta-analysis; JL and YG provided statistical advice and input; XM drafted the protocol. DZ and JT reviewed the protocol and provided critical feedback. All authors approved the article in its final form.

## Supplementary Material

Supplemental Digital Content
