# Supplementary material for: The association between body mass index and the risk of different gastrointestinal cancers: A protocol for an overview of systematic reviews
Source: Medicine (Baltimore). 2018 Nov 9;97(45):e13181. doi: 10.1097/MD.0000000000013181 (PMC6250501; doi:10.1097/MD.0000000000013181)
Supplement: Supplemental Digital Content [file medi-97-e13181-s001.docx]

**The association between body mass index and the risk of different gastrointestinal cancers: a protocol for an overview of** **systematic reviews**

Xueni Ma, MD^a^, Ya Gao, MM^b^, Jipin Li, MD^a^, Dekui Zhang, PhD ^c^* and Jinhui Tian, PhD ^b*^

Supplementary 1 searching strategy of Pubmed

#1 "Obesity"[Mesh] OR Obesity[Title/Abstract] OR Obesities[Title/Abstract]

#2 "Pediatric Obesity"[Mesh] OR "Pediatric Obesity"[Title/Abstract] OR "Childhood Obesity"[Title/Abstract] OR "Childhood Onset Obesity"[Title/Abstract] OR "Child Obesity"[Title/Abstract] OR "Childhood Overweight"[Title/Abstract] OR "Childhood Overweights"[Title/Abstract] OR "Infant Obesity"[Title/Abstract] OR "Infant Overweight"[Title/Abstract] OR "Infantile Obesity"[Title/Abstract] OR "Adolescent Obesity"[Title/Abstract] OR "Adolescent Overweight"[Title/Abstract]

#3 "Obesity, Abdominal"[Mesh] OR "Abdominal Obesities"[Title/Abstract] OR "Abdominal Obesity"[Title/Abstract] OR "Central Obesity"[Title/Abstract] OR "Central Obesities"[Title/Abstract] OR "Visceral Obesity"[Title/Abstract] OR "Visceral Obesities"[Title/Abstract]

#4 "Obesity, Morbid"[Mesh] OR "Morbid Obesities"[Title/Abstract] OR "Severe Obesities"[Title/Abstract] OR "Severe Obesity"[Title/Abstract] OR "Morbid Obesity"[Title/Abstract]

#5 "Obesity, Metabolically Benign"[Mesh] OR "Metabolically Healthy Obesity"[Title/Abstract] OR "Metabolically Benign Obesity"[Title/Abstract]

#6 "Abdominal Fat"[Mesh] OR "Abdominal Fats"[Title/Abstract] OR "Abdominal Adipose Tissue"[Title/Abstract] OR Fat[Title/Abstract] OR Fats[Title/Abstract] OR Fatness[Title/Abstract]

#7 "Adiposity"[Mesh] OR Adipose[Title/Abstract] OR Adiposis[Title/Abstract]

#8 "Overweight"[Mesh] OR Overweight[Title/Abstract] OR Overweights[Title/Abstract] OR Weight[Title/Abstract] OR "Excess body weight"[Title/Abstract]

#9 "Body Size"[Mesh] OR "Body Size"[Title/Abstract] OR "Body Sizes"[Title/Abstract]

#10 "Body Mass Index"[Mesh] OR "Quetelet Index"[Title/Abstract] OR "Quetelet's Index"[Title/Abstract] OR "Quetelets Index"[Title/Abstract] OR BMI[Title/Abstract]

#11 #1 OR #2 OR #3 OR #4 OR #5 OR #6 OR #7 OR #8 OR #9 OR #10

#12 "Meta-Analysis" [Publication Type] OR "Meta-Analysis as Topic"[Mesh] OR "Network Meta-Analysis"[Mesh] OR Meta-Analysis[Title/Abstract] OR "Meta Analysis"[Title/Abstract] OR Meta-Analyses[Title/Abstract] OR "Meta Analyses"[Title/Abstract] OR "gathering analysis"[Title/Abstract] OR "Network Meta-Analyses"[Title/Abstract] OR "Network Meta Analysis"[Title/Abstract] OR "Network Meta Analyses"[Title/Abstract] OR "Mixed Treatment Meta-Analysis"[Title/Abstract] OR "Mixed Treatment Meta-Analyses"[Title/Abstract] OR "Multiple Treatment Comparison Meta-Analysis"[Title/Abstract] OR "Multiple Treatment Comparison Meta Analysis"[Title/Abstract]

#13 "Systematic evaluation"[Title/Abstract] OR "Systematic assessment"[Title/Abstract] OR "Systematic review"[Title/Abstract] OR "Systematic reviews"[Title/Abstract] OR "System evaluation"[Title/Abstract] OR "System Assessment"[Title/Abstract] OR "Systemic review"[Title/Abstract] OR "Systemic reviews"[Title/Abstract]

#14 #12 OR #13

#15 #11 AND #14
